# Supplementary figures and images for: Host Gut Motility Promotes Competitive Exclusion within a Model Intestinal Microbiota
Source: PLoS Biol. 2016 Jul 26;14(7):e1002517. doi: 10.1371/journal.pbio.1002517 (PMC4961409; doi:10.1371/journal.pbio.1002517)

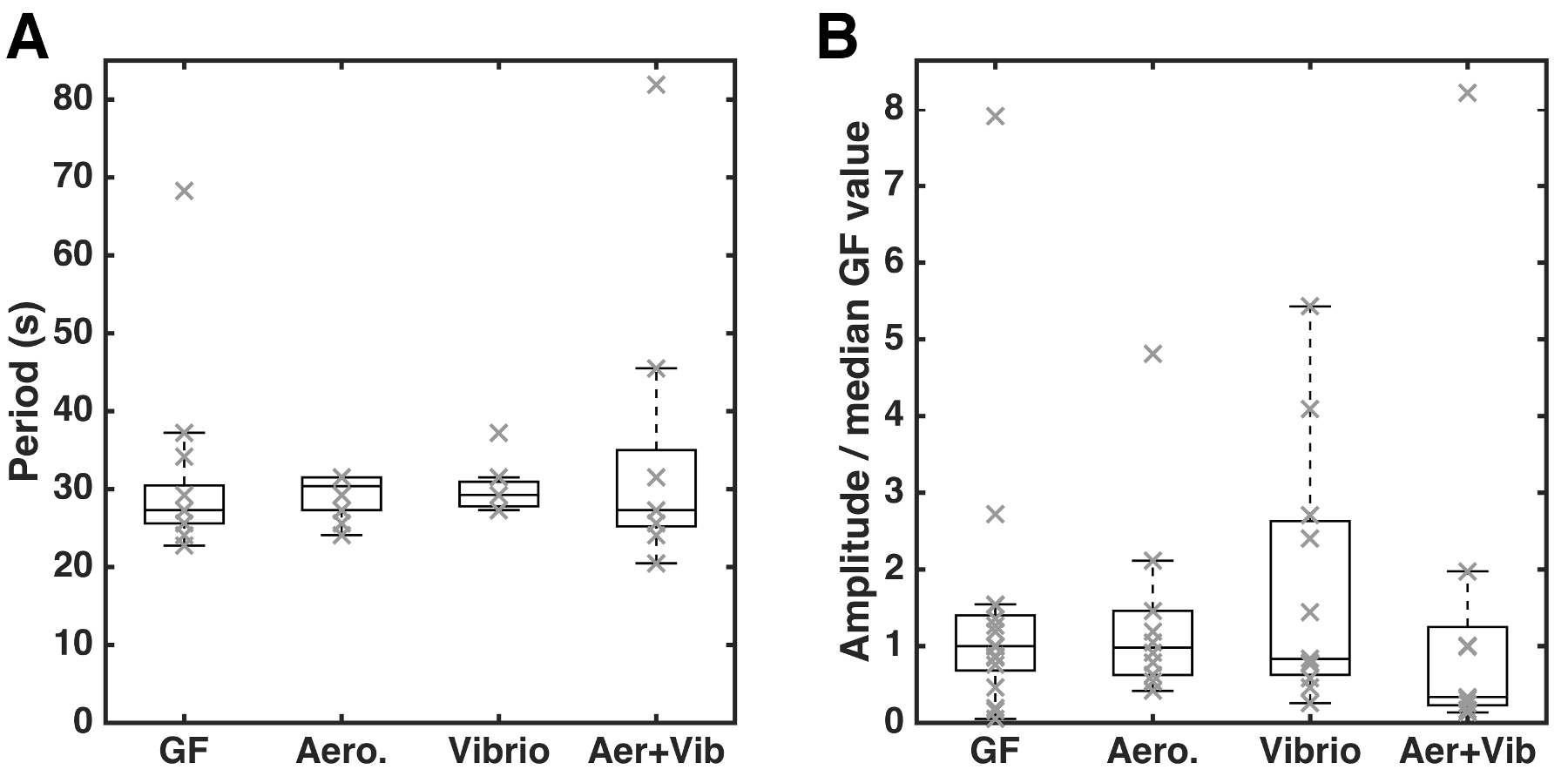

Supplement: S4 Fig — GF = germ-free; Aero = mono-association with Aeromonas from 4 dpf; Vibrio = mono-association with Vibrio from 4 dpf; Aer+Vib = mono-associated with Aeromonas at 4 dpf and challenged with Vibrio at 5 dpf. (A) The characteristic period of gut motility, identified as the inverse of the frequency of the peak signal in a Fourier spectrum of gut motion amplitudes, averaged over all positions. All conditions give very similar periodicity of gut motion. (B) The characteristic amplitude of gut motility, identified as magnitude of the peak signal in a Fourier spectrum of gut motion amplitudes. There is considerable variability between fish clutches, and so the amplitudes are normalized by the median of the germ-free fish in each batch. All conditions show large variance, with no significant difference evident between the various conditions. In A and B, gray X’s are from individual fish; boxes indicate the first to third quartiles, and the horizontal bars in boxes indicates the median value. Underlying data for A and B are provided in S1 Data. (TIF) [file pbio.1002517.s005.tif]
