# Supplementary material for: Host Gut Motility Promotes Competitive Exclusion within a Model Intestinal Microbiota
Source: PLoS Biol. 2016 Jul 26;14(7):e1002517. doi: 10.1371/journal.pbio.1002517 (PMC4961409; doi:10.1371/journal.pbio.1002517)
Supplement: S1 Text — (PDF) [file pbio.1002517.s017.pdf]

SUPPORTING TEXT

Host Gut Motility Promotes Competitive Exclusion within a Model Intestinal Microbiota

Travis J. Wiles, Matthew Jemielita, Ryan P. Baker, Brandon H. Schlomann, Savannah L. Logan, Julia Ganz, Ellie Melancon, Judith S. Eisen, Karen Guillemin, Raghuveer Parthasarathy

Stochastic collapse model

We describe a simple model of growth and collapse behavior and examine its predictions for population sizes. We also fit the model to experimental data on bacterial abundance.

1 The model

Consider a species with population *N* at time *t* that exhibits logistic growth, with growth rate *r* and carrying capacity *K*:

$$dN = rN\left(1-\frac{N}{K}\right)dt \quad , \qquad \text{[Equation 1]}$$

We superimpose on these dynamics events in which the population collapses to a value *f* times its pre-collapse value, where *f* is between 0 and 1, after which it resumes logistic growth. We model the timing of the collapses as a Poisson process: collapses are uncorrelated and stochastic, occurring with a probability per unit time p_c. Formally, one can write this as a stochastic differential equation:

$$dN = rN\left(1-\frac{N}{K}\right)dt-(1-f)N\,dM \quad , \qquad \text{[Equation 2]}$$

where *dM* is a Poisson process of unit step. (In other words, *dM* =1 with probability $p_c dt$, and *dM* = 0 with probability 1 - $p_c dt$.) *N dM* refers to *N* immediately before the collapse. An illustration of the roles of the parameters *r*, *K*, *f*, and p_c is provided in Figure 5. As noted in the main text, this model is not new; it has been invoked and studied in many ecological contexts [S1]. However, the particular treatment presented here is, to the best of our knowledge, novel, especially with respect to determining relevant parameters for fits to experimental data. We determine statistical properties of the model using numerical simulations. For infinite carrying capacity, these properties can be calculated analytically, but for the biologically relevant case of finite carrying capacity, exact solutions do not at present exist.

2 Simulations

The model described above is simple to simulate by numerical integration, which yields the population x_t at time t. Simulation code is provided as Supporting Material (S1 Program, written in MATLAB). Two typical x_t are shown in Figure ST1, with parameters as noted in the caption.

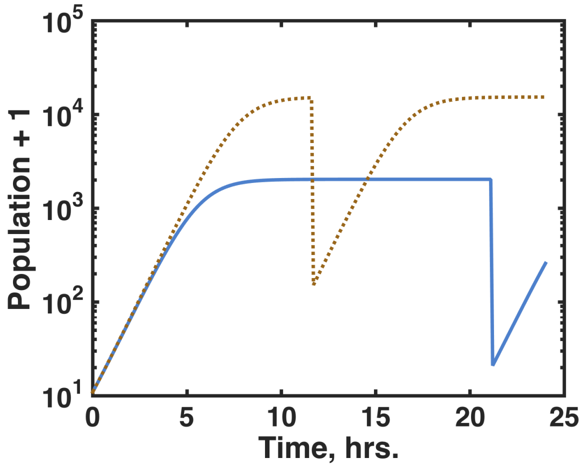

Figure ST1. Two simulated populations exhibiting stochastic collapses, with $f = 10^{-2}$, $p_c = 0.05$ hr $^{-1}$, $r = 1$ hr $^{-1}$, and K drawn from a log-normal distribution with mean 10^4 and a standard deviation of half a decade. (We plot the population plus one so that zero values are evident on the logarithmic scale.)

The model has four parameters, r, K, f, and p_c, and a boundary condition set by x_0 (the initial population). The value of x_0 is irrelevant for the experimental conditions considered: the populations start from a small value and grow rapidly. In our simulations x_0 is taken to be 10.

The growth rate, r, is known from measurements. Moreover, the model dynamics are fairly insensitive to r, since the experimental timescales of ~10 hours are considerably larger than the timescale set by the growth rate ($1/r$ ~ 1 hour).

The key determinants of the population statistics, therefore, are the collapse properties (p_c and f) and the carrying capacity, K. The carrying capacity may exhibit considerable variation between fish. Typically, the final populations of *Aeromonas* in mono-associations are found to be approximately log-normally distributed (Figure ST2), as is commonly the case for species abundances, and so in simulations we draw K from log-normal distributions. In other words, $\log_{10}(K)$ for a given simulation is drawn from a

Gaussian distribution with some mean value and standard deviation σ_K, where σ_K is typically 0.5, discussed further below. We note that in the absence of collapse (e.g., p_c = 0 or f=1) this model is completely deterministic, and the variance in final bacterial populations between fish is solely due to the variance in K.

For particular parameter values, we simulate many instances of the above dynamics (typically 1,000 to 10,000) and examine the statistical properties of the final population, x_t, assessed at t = 24 hours. For the values used in Figure ST1 above, for example, the mean and standard deviation of the final x_t are $(6.8 \pm 18.5) \times 10^3$. The distributions span orders of magnitude, including zero, so it is useful to consider the mean and standard deviation of $\log_{10}(x_t+1)$, similar to a geometric mean. For these parameters, this gives a mean and standard deviation of $\log_{10}(x_t+1)$ of 2.0 ± 1.8. We will define y as

$$y = \log_{10}(x_t + 1) \quad , \qquad \text{[Equation 3]}$$

for notational simplicity.

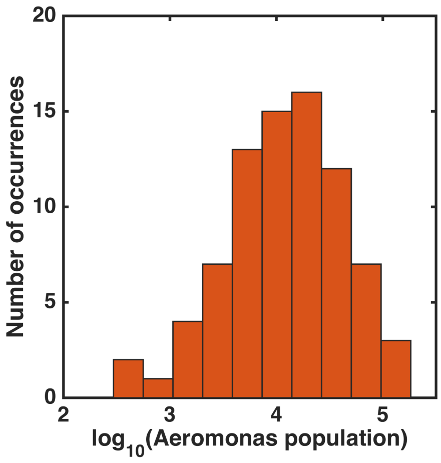

Figure ST2. Histogram of the final population of *Aeromonas* mono-associated with larval zebrafish at 4 dpf and assessed at days 5, 6, or 7 dpf by plating of dissected gut contents and counting of colony forming units.

3 Parameters and Fits

3.1 Dependence on p_c and f

We can vary the model parameters to determine the relationship between the mean and the variance of the final population, which will allow direct comparison between our model and measurements of bacterial abundance (e.g., Figure 1). The dependence of the mean and standard deviation (std.) of y on p_c and f is plotted in Figure ST3. We can intuitively understand its behavior: for small p_c or f near 1, the

properties of x_t are largely set by the mean and variance of the carrying capacity. However, as p_c increases (or f decreases), the mean of x_t decreases, because larger collapses are more likely to occur, and the standard deviation of x_t increases, because the stochastic collapses play a more significant role in the dynamics. For still larger p_c (or smaller f), the final population becomes more uniformly small, because the population is dominated by very frequent collapses and cannot grow appreciably.

Treating f as a random, rather than a fixed, parameter has little effect on the behavior of the model. Drawing f from a beta distribution, chosen because it is continuous, spans [0, 1], and has two parameters that can be mapped onto a mean and variance, gives the curve shown in Figure ST4. The mean collapse magnitude is chosen over the same range as f in Figure ST3, and for each mean f, several f values are drawn from a beta distribution with standard deviations relative to the mean spanning [0, 0.8]. All the resulting population characteristics are plotted in Figure ST4; the resulting curve is nearly identical to that of Figure ST3.

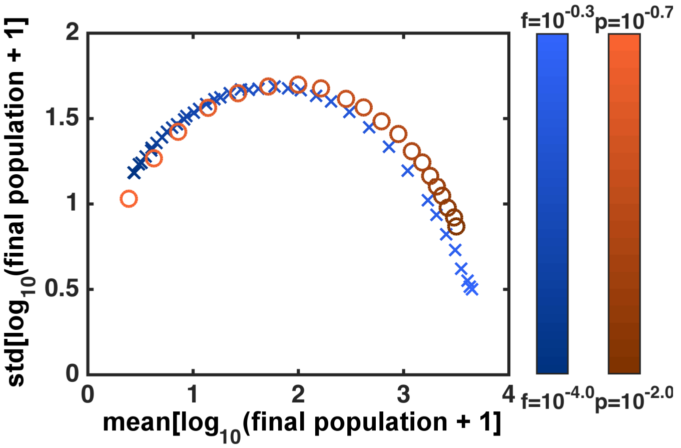

Figure ST3. The mean and standard deviation of simulated populations at t = 24 hrs., with r = 0.8 hr $^{-1}$ and K drawn from a log-normal distribution with mean 10^4 and a standard deviation of half a decade.
Blue crosses: p_c is fixed at = 0.1 hr^{-1}, and f varies between 10^{-4} and $10^{-0.3}$.
Red circles: f is fixed at 10^{-2} and p_c varies between 10^{-2} and $10^{-0.7}$ hr^{-1}.
Each point is calculated from 10,000 simulated runs.

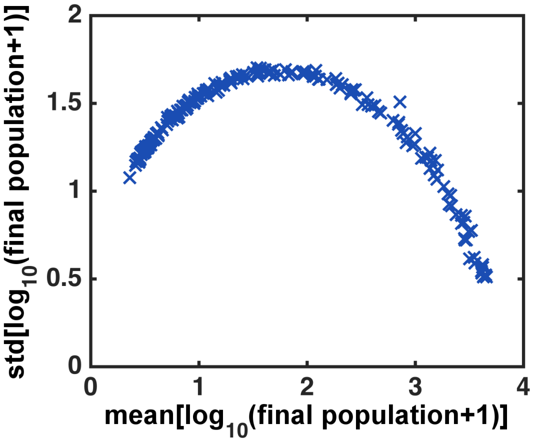

Figure ST4. The mean and standard deviation of simulated populations at t = 24 hrs., with r = 1 hr^{-1} and K drawn from a log-normal distribution with mean 10^4 and a standard deviation of half a decade. The collapse probability p_c is fixed at = 0.1 hr^{-1}, and f is drawn from a beta distribution with mean between 10^{-4} and $10^{-0.3}$, and relative standard deviation between 0 and 80%. Each point is calculated from 1,000 simulated runs.

Remarkably, at fixed K, nearly identical curves result from varying either p_c or f (Figure ST3), suggesting that at least over the parameter ranges and timescales relevant to our experiments, these two parameters can be subsumed into one effective variable. Considering particular values of mean(y) and std(y), where y is the logarithm of the population as defined above, we can search for the best-fit values of (p_c, f), i.e., the parameters that minimize the squared Euclidean distance, χ^2, between the measured and simulated (mean(y), std(y)). Using, for concreteness, the values determined from gut dissection and plating experiments of *Aeromonas* abundance 24 hours after challenge by *Vibrio*, namely (mean(y), std(y)) = (1.68 ± 0.34, 1.50 ± 0.24), we find, as expected, the best-fit contours describe a curve in the (p_c, f) space (Figure ST5a). Empirically, we find that this curve is represented by $-p_c \log_{10}(f) \approx$ constant (Figure ST5b).

Fitting experimental data to this model of logistic growth with stochastic collapses reduces, therefore, to a two parameter fit to the carrying capacity, K, and a parameter describing the collapse properties, denoted as z:

$$z = -p_c \log_{10}(f) \ , \qquad \text{[Equation 4]}$$

To the best of our knowledge, this effective collapse of the two stochastic parameters into one effective parameter, z, has not been previously reported. We do not have a mathematically exact theory for its occurrence, but simply present it as an empirical result from our numerical simulations.

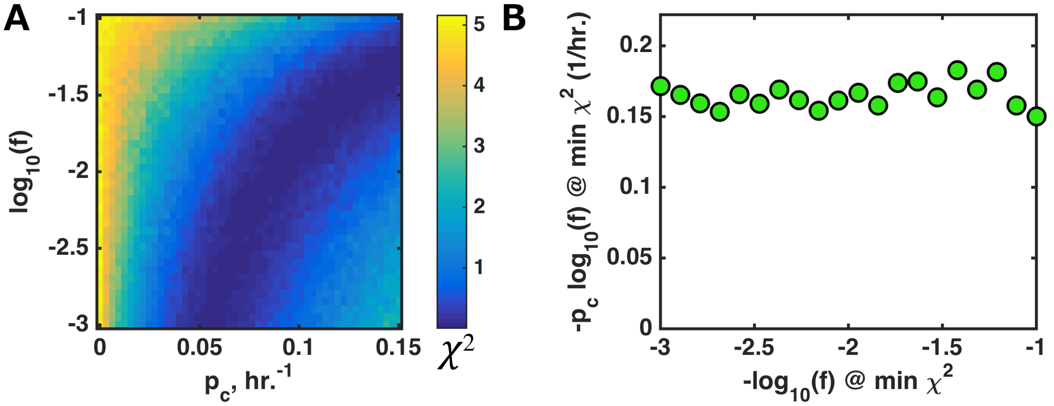

Figure ST5. (A) Squared distance, χ^2, between the measured and simulated (mean(y), std(y)) for values derived from *Aeromonas* abundance 24 hours after challenge by *Vibrio*, namely (mean(y), std(y)) = (1.68 ± 0.34, 1.50 ± 0.24), as a function of model parameters p_c and f. The carrying capacity is drawn from a log-normal distribution with mean $10^{3.7}$ and standard deviation 0.5 decades. At each value of (p_c, f), 1000 runs are simulated to determine mean(y) and std(y). The optimal parameters (darkest blue) sweep out a curve in the parameter space. (B) The optimal p_c and f are related by $-p_c \log_{10}(f) \approx$ constant over the range of parameters examined.

3.2 Parameter fits: *Aeromonas* challenged by *Vibrio*

Again using the *Aeromonas* 24-hour post-challenge abundance data (Figure 1), (mean(y), std(y)) = (1.68 ± 0.34, 1.50 ± 0.24), contours of χ^2 are shown in Figure ST6. The best-fit parameter values are:

$z = -p_c \log_{10}(f) = 0.13 \pm 0.05 \text{ hr}^{-1}$,

$\log_{10}(K) = 3.2 \pm 0.5$

In the simulations, K is drawn from a log-normal distribution with width 0.5 decades; the fit is insensitive to this width, since the variance in the final population is much greater than 0.5. The uncertainties in z and K are estimated from simulations spanning the experimental uncertainties in mean(y) and std(y).

In the main text, we compare these plating-derived measures of the collapse parameters p_c and f to those determined from live imaging.

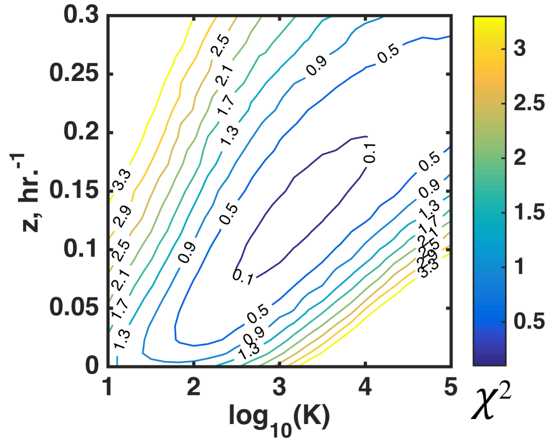

Figure ST6. Contours of χ^2, the distance between simulated (mean(y), std(y)) and the measured value from di-association experiments (1.68, 1.50), for a range of z and K. The fit has a clear minimum at z = 0.13 hr $^{-1}$ and $\log_{10}(K)$ = 3.2.

3.3 Parameter fits: *Aeromonas* alone

Similarly, we can determine the parameter values that best match *Aeromonas* mono-association data, (mean(y), std(y)) = (4.1 ± 0.08, 0.61 ± 0.05), where these values are from plating data at both 5 and 6 days post-fertilization. Because std(y) is low, i.e., the data map onto the lower right corner of the curve of Figures ST3-4, it is unclear whether the variance in y is due mainly to variance in K or to the stochasticity of collapses, and we have no independent measure of the variance in K. Considering K drawn from log-normal distributions of various widths, we find best-fit values of $z = -p_c\log_{10}(f)$ spanning roughly z = 0.01 ± 0.01 hr^{-1}, i.e., z is poorly constrained. Contours of χ^2 are shown in Figure ST7. Despite this uncertainty, K is well-constrained to be approximately $\log_{10}(K)$ = 4.2 ± 0.1. The significance of this is discussed in the main text.

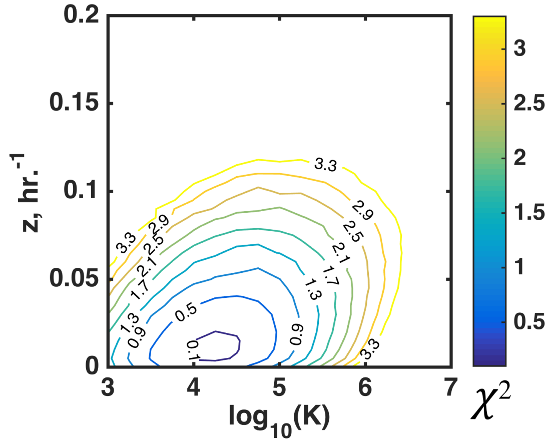

Figure ST7. Contours of χ^2, the squared distance between simulated (mean(y), std(y)) and the measured value from mono-association experiments (4.1, 0.6), for a range of z and K, with K drawn from log-normal distributions of width 0.1 decades.

SUPPORTING REFERENCES

S1. Hanson FB, Tuckwell HC. Logistic growth with random density independent disasters. Theoretical population biology. 1981;19(1):Pages 1-18.
